# The African Esophageal Cancer Consortium: A Call to Action

**DOI:** 10.1200/JGO.17.00163

**Published:** 2018-01-25

**Authors:** Katherine Van Loon, Michael M. Mwachiro, Christian C. Abnet, Larry Akoko, Mathewos Assefa, Stephen L. Burgert, Steady Chasimpha, Charles Dzamalala, David E. Fleischer, Satish Gopal, Prasad G. Iyer, Bongani Kaimila, Violet Kayamba, Paul Kelly, Maria E. Leon, Christopher G. Mathew, Diana Menya, Daniel Middleton, Yohannie Mlombe, Blandina T. Mmbaga, Elia Mmbaga, Gift Mulima, Gwen Murphy, Beatrice Mushi, Ally Mwanga, Amos Mwasamwaja, M. Iqbal Parker, Natalie Pritchett, Joachim Schüz, Mark D. Topazian, Russell E. White, Valerie McCormack, Sanford M. Dawsey

**Affiliations:** **Katherine Van Loon**, University of California, San Francisco, San Francisco, CA; **Michael M. Mwachiro**, **Stephen L. Burgert**, and **Russell E. White**, Tenwek Hospital, Bomet; **Diana Menya**, Moi University, Eldoret, Kenya; **Christian C. Abnet**, **Gwen Murphy**, **Natalie Pritchett**, and **Sanford M. Dawsey**, National Cancer Institute, Bethesda, MD; **Larry Akoko**, **Elia Mmbaga**, **Beatrice Mushi**, and **Ally Mwanga**, Muhimbili University of Health and Allied Sciences, Dar es Salaam; **Blandina T. Mmbaga** and **Amos Mwasamwaja**, Kilimanjaro Clinical Research Institute, Moshi, Tanzania; **Mathewos Assefa**, Addis Ababa University, Addis Ababa, Ethiopia; **Steady Chasimpha** and **Charles Dzamalala**, Queen Elizabeth Central Hospital; **Charles Dzamalala**, **Satish Gopal**, **Bongani Kaimila**, and **Yohannie Mlombe**, University of Malawi College of Medicine, Blantyre; **Gift Mulima**, Kamuzu Central Hospital, Lilongwe, Malawi; **David E. Fleischer**, Mayo Clinic, Phoenix, AZ; **Satish Gopal**, University of North Carolina, Chapel Hill, NC; **Prasad G. Iyer** and **Mark D. Topazian**, Mayo Clinic, Rochester, MN; **Violet Kayamba** and **Paul Kelly**, University of Zambia, Lusaka, Zambia; **Paul Kelly**, Queen Mary University of London; **Christopher G. Mathew**, King’s College London, London, United Kingdom; **Maria E. Leon**, **Daniel Middleton**, **Joachim Schüz**, and **Valerie McCormack**, International Agency for Research on Cancer, Lyon, France; **Christopher G. Mathew**, University of the Witwatersrand, Johannesburg; and **M. Iqbal Parker**, University of Cape Town, Cape Town, South Africa.

## Abstract

Esophageal cancer is the eighth most common cancer worldwide and the sixth most common cause of cancer-related death; however, worldwide incidence and mortality rates do not reflect the geographic variations in the occurrence of this disease. In recent years, increased attention has been focused on the high incidence of esophageal squamous cell carcinoma (ESCC) throughout the eastern corridor of Africa, extending from Ethiopia to South Africa. Nascent investigations are underway at a number of sites throughout the region in an effort to improve our understanding of the etiology behind the high incidence of ESCC in this region. In 2017, these sites established the African Esophageal Cancer Consortium. Here, we summarize the priorities of this newly established consortium: to implement coordinated multisite investigations into etiology and identify targets for primary prevention; to address the impact of the clinical burden of ESCC via capacity building and shared resources in treatment and palliative care; and to heighten awareness of ESCC among physicians, at-risk populations, policy makers, and funding agencies.

Esophageal cancer is the eighth most common cancer worldwide and the sixth most common cause of cancer-related death; however, worldwide incidence and mortality rates do not reflect the geographic variations in the occurrence of this disease.^1^ More than 80% of cases and deaths from esophageal cancer occur in developing countries, and geographically defined high-incidence regions are a striking and distinguishing characteristic of this malignancy. In contrast to the economically developed world, where the dominant subtype of esophageal cancer is adenocarcinoma, esophageal squamous cell carcinoma (ESCC) is the dominant subtype in high-incidence areas, including Africa.

High-incidence areas for ESCC have been known and studied for years in northern China, northeastern Iran, southern South America, and South Africa.^2-9^ Another geographically defined high-incidence area is the eastern corridor of Africa. The common occurrence of ESCC along this corridor was documented more than a half-century ago and mapped as far back as 1969.^10^ More recent evidence has demonstrated that this region—extending from Ethiopia to South Africa—remains disproportionately impacted by the high incidence of ESCC.^11,12^ In stark contrast, esophageal cancer is much less common in Western or Northern Africa^1^ (Fig 1).

**Fig 1:**
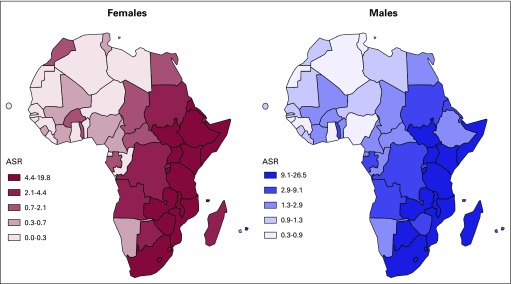
National-level map of age-standardized incidence rate (ASR) of esophageal squamous cell carcinoma in women and men (Arnold et al^13^).

Reflecting the significant impact of ESCC in the developing world, the US National Cancer Institute (NCI) and the International Agency for Research on Cancer (IARC) convened an International Tumor Workshop on Esophageal Squamous Cell Carcinoma in September 2016.^14^ Whereas this meeting was attended by international experts who represented multiple high-incidence regions throughout the developing world, a substantial focus was on the emerging data and unmet needs related to the high incidence of ESCC in sub-Saharan Africa. At this meeting, a critical mass of African investigators and their international partners initiated the African Esophageal Cancer Consortium (AfrECC) to facilitate research collaborations in environmental, molecular, and genetic epidemiology; early detection, clinical management, treatment, and palliation; capacity building; and interventions to reduce the burden of ESCC in sub-Saharan Africa.

## CURRENT AfrECC SITES

**Fig 2:**
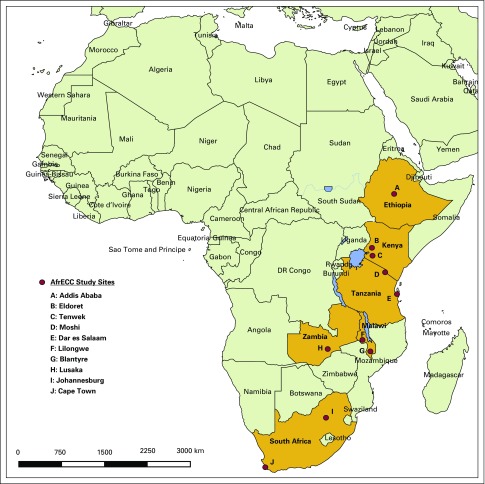
Current participant sites in the African Esophageal Cancer Consortium (AfrECC).

During the past few years, several groups have initiated epidemiologic and molecular studies of ESCC at sites in sub-Saharan Africa, including Dar es Salaam, Tanzania (Muhimbili University of Health and Allied Sciences, Ocean Road Cancer Institute, and University of California, San Francisco); Eldoret, Kenya (Moi University and IARC); Bomet, Kenya (Tenwek Hospital and NCI); Moshi, Tanzania (Kilimanjaro Clinical Research Institute and IARC); Lilongwe, Malawi (The University of North Carolina Project-Malawi and NCI); Blantyre, Malawi (Queen Elizabeth Central Hospital and IARC); Lusaka, Zambia (Tropical Gastroenterology and Nutrition Group, University of Zambia); Addis Ababa, Ethiopia (Addis Ababa University and IARC); and Johannesburg and Cape Town, South Africa (University of the Witwatersrand and University of Cape Town). These groups have now joined together in AfrECC (Fig 2) to share expertise, infrastructure, and resources and to ensure that all present and future participant sites in sub-Saharan Africa fully benefit from our collaborative work. During regular conference calls, participants review ongoing activities, identify opportunities and challenges, and plan for the coordination of multisite efforts. In May 2017, AfrECC members convened in East Africa and conducted visits to several AfrECC sites to learn from each other and facilitate closer collaboration. In the future, we aim to establish formal membership guidelines and pursue shared research funding to optimize the impact of our resources and efforts to reduce mortality and suffering from ESCC in this region.

## CURRENT AfrECC PRIORITIES

### Implement Coordinated Multisite Investigations Into the Etiology of ESCC in East Africa and Identify Targets for Primary Prevention

Studies from China and Iran, as well as from the United States, Europe, and Japan, demonstrate considerable etiologic heterogeneity for ESCC, with evidence that major risk factors in one population may play a more limited role in other populations. Whereas etiologic and genetic studies of esophageal cancer in Asian populations have been relatively extensive, the high-incidence region along the eastern corridor of Africa remains largely unstudied. We recently conducted an extensive review to assess whether ESCC risk factors that have been established or are likely in other parts of the world are also present in the African context. Whereas many were identified, most have not been carefully studied in Africa.^15^ Moreover, in a number of East African populations, we observed a disproportionate number (approximately 20%) of patients who were diagnosed at younger than 40 years old.^12,16-19^ This high incidence of ESCC in patients younger than 40 years, as well as the geographic variation associated with this diagnosis, suggests plausible roles for unique environmental, infectious, and/or genetic risk factors in this region. Identification of environmental, molecular, and genetic factors, as well as possible interactions, that contribute to the high incidence of this disease along the eastern corridor of Africa will be necessary to inform prevention and early detection strategies in this region.

Case control studies will be a primary method of etiologic research in this setting. A full case control study has completed accrual in Dar es Salaam, Tanzania,^17^ and smaller studies from Zambia^20^ and Ethiopia^21^ have been published. NCI and IARC are supporting several parallel ongoing case control studies at other AfrECC participant sites, collectively known as the ESCCAPE case control studies.^22^ At the three sites that began studies in 2016 and 2017, questionnaires have been harmonized and data are being collected electronically using a mobile health application, which helps standardize the data across sites, allows centralized and remote coordination, and improves real-time quality control. Whereas each individual site has the potential to accrue hundreds of patients, no single site is likely to identify the etiologic factors for the development of ESCC throughout the region, and no single study has the potential to confirm associations; thus, multisite collaborations with harmonized study instruments and protocols are needed to allow for comparison and validation of findings. Ultimately, we aim to merge data from multiple study sites to compare and contrast the etiological effects of lifestyle and environmental factors on ESCC across this region. Extensive questionnaire data and saliva and/or blood specimens for future DNA analyses are being collected from all study participants, and multisite genome-wide association studies, such as those previously conducted to investigate this disease in China,^23^ are planned.

Case control studies will seek to identify exposures that are implicated in ESCC in Africa; however, many associations will necessarily rely on self-reported data, as biomarker-based assessments are typically not feasible as a result of the severe alteration of the exposure—for example, diet—caused by the disease. Thus, the consortium is also undertaking detailed ecologic and cross-sectional descriptive studies of specific exposures, such as hot beverages^24^ and micronutrient deficiencies,^25^ to understand major exposure sources, levels, and exposed population groups.

In addition, establishment of ESCC tumor biorepositories for additional genomic profiling is underway. Mutation of the tumor suppressor gene, *TP53*, has been previously reported as the most frequent genetic alteration in ESCC in other settings,^26,27^ with mutation profiles that are known to vary across geographic areas^28,29^; however, a recent whole-exome sequencing and RNA sequencing analysis of 59 ESCC tumors from Malawi reported a high proportion of tumors without *TP53* mutations, as well as a tumor mutation signature^30^ that is possibly consistent with an unknown carcinogenic exposure.^31^ This sequencing study from Malawi and case series from South Africa, Zambia, and Kenya have not supported an etiologic role for human papillomavirus,^20,32-34^ and, with rare exception,^20,35^ there is no evidence of an association of ESCC with HIV, either in time trends or in case control data^36^; however, a search for other possible infectious etiologies remains an area of active investigation.

Finally, we recognize that the sustainability and success of our research is ultimately dependent on the efforts of clinical and research colleagues in Africa, and we are committed to the provision of training in research methodology and longitudinal mentoring to enhance and promote cancer research capabilities in the region.

### Address the Impact of the Clinical Burden of Esophageal Cancer Through Capacity Building and Shared Resources in Treatment and Palliative Care

As in other low- and middle-income countries, patients with ESCC in eastern Africa present with advanced disease with symptoms of dysphagia and obstruction and resultant malnutrition. Patients with ESCC are readily identifiable in surgical and medical hospital wards, profoundly wasted and holding spittoon cups to manage the secretion of saliva. The suffering of these patients is profound and weighs heavily on the minds of the physicians and nurses who care for them, many of whom share sentiments of helplessness and hopelessness because of the lack of available options for early detection, treatment or palliation.

Meanwhile, at Tenwek Hospital, a community-based referral hospital in Bomet, Kenya, where ESCC accounts for 35% of all tumors, surgeons have deployed more than 2,000 self-expanding metal stents (SEMSs) for patients who present with esophageal obstruction. In a review of 1,000 patients who received SEMSs, this technology was demonstrated to be safe and feasible for the palliation of dysphagia symptoms related to ESCC in resource-limited settings^37^; however, at most other sites in eastern Africa, the retail price for imported SEMSs is prohibitive for patients, a barrier that is compounded by the low socioeconomic status and insufficient health insurance of these patients. As a result of financial barriers, the poor distribution of relatively inexpensive SEMSs, and inadequate endoscopy resources and training, palliative stenting is not widely available in eastern Africa. AfrECC is pursuing several options to support advocacy for equal and fair pricing and access to SEMS for the region, as well as capacity-building activities to support endoscopic training, including SEMS placement.

A recent report from China also suggests that steady, incremental progress may eventually yield high-impact programs for ESCC screening and earlier detection, which can significantly reduce mortality.^38^ With increased advocacy and awareness about the high incidence of ESCC impacting the region, we anticipate that earlier detection may yield an eventual trend toward increased numbers of patients presenting with tumors that are amenable to curative therapies, including endoscopic therapy, chemoradiation, and/or surgery. We aim to support the development of standardized treatment protocols that are relevant to the available resources within the region. The formation of surgical training partnerships will also facilitate training and the establishment of proficiencies in complex esophagectomy procedures. Finally, implementation strategies are needed to enhance and measure the acceptability, feasibility, and sustainability of each of these interventions, and long-term monitoring is needed to evaluate their effects on clinical outcomes.

### Heighten Awareness of ESCC Among physicians, At-Risk Populations, Policy Makers, and Funding Agencies

**Fig 3:**
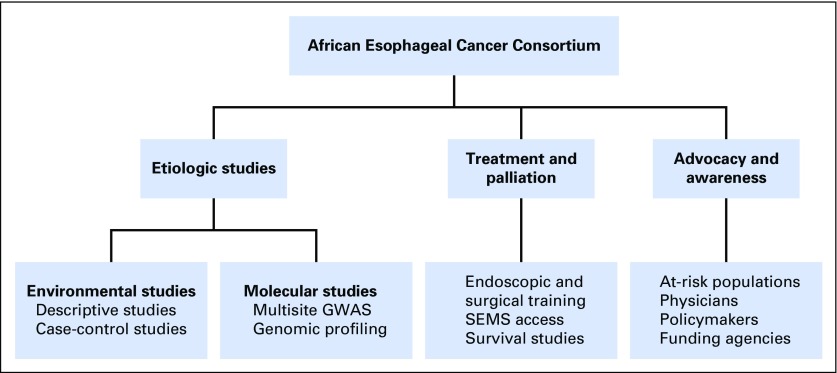
Priority activities of the African Esophageal Cancer Consortium. GWAS, genome-wide association study; SEMS, self-expanding metal stent.

In the absence of the capacity to provide treatment or palliation for patients with ESCC at most sites throughout eastern Africa, individuals who develop symptoms of dysphagia are commonly reticent to seek care, and there is a pervasive perception that going to the hospital with symptoms of dysphagia means certain death. Sadly, this perception is currently not far from reality, with patients routinely presenting with extremely advanced disease and at risk for rapid deterioration; however, extrapolating from other diseases, increased awareness and education among at-risk populations and physicians may result in earlier presentations for clinical care, earlier diagnosis, and referrals to centers that are equipped to provide the necessary treatment or palliation. In addition, increased advocacy and awareness among local ministries of health and international funding agencies is critical for the provision of resources to increase access to relevant therapies and to support the necessary research to drive the development of evidence-based policies for prevention and palliation. The priority activities of the consortium are summarized in Figure 3.

## CONCLUSION

In May 2017, the World Health Assembly endorsed a set of measures to improve and scale up access to prevention, early diagnosis, prompt and accessible treatment, and palliative care for cancer.^39^ Member States called on WHO to promote access for all people to affordable cancer diagnosis and treatment and to provide countries with technical guidance to identify and implement priority cancer control interventions. WHO member states committed to ensuring adequate resources to support the implementation of national cancer control plans and to strengthen health systems to provide early diagnosis and treatment services for all patients with cancer. ESCC is one of the most prevalent and deadly cancers that afflicts eastern and southern Africa, and it is certainly one that puts vulnerable populations at risk for great morbidity and catastrophic health expenditures.

The task of scaling up care for ESCC within African health systems that are already fraught with challenges has been dismissed by some as impractical and unattainable because of the deadly nature of this disease. Whereas we acknowledge the challenges, our shared view is that this is the very attribute that makes this work urgent and imperative. We know from the evolution of the HIV epidemic that etiologic understanding, palliation, health system strengthening, capacity building, and the gradual implementation of effective prevention, early detection, and treatment interventions can provide a path forward, even when faced with the most daunting of diseases.

Although international attention to the tremendous burden of this disease is nascent, there is burgeoning high-quality work in progress at multiple sites in Kenya, Tanzania, Malawi, Zambia, Ethiopia, and South Africa, with the development of etiologic studies, biospecimen repositories, genome-wide association studies, and clinical training partnerships. Looking forward, we aim to enhance the ongoing efforts through increased collaboration across sites and strengthened international partnerships. Our call to action aims to raise awareness of the tremendous impact of this deadly disease on an already fragile region and to mobilize others to become involved.
